# Optimizing the Production of a Condensed Tannin-Based Bioflocculant for Toxic Metal Remediation

**DOI:** 10.1021/acsomega.6c04045

**Published:** 2026-09-13

**Authors:** Sara Raissa Lobato Ferreira, Jéssica Lyssa Araújo, Camilla Mariane Moura de Souza, Carlos Gabriel da Silva de Souza, Kleber Raimundo Freitas Faial, Neirivaldo Cavalcante da Silva, Nilton Akio Muto

**Affiliations:** † Center for Valorization of Bioactive Compounds of the Amazon (CVACBA), Guamá Science and Technology Park (PCT Guamá), Federal University of Pará, Perimetral da Ciência Avenue, s/n, Espaço Inovação Building, Guamá, 66075-750 Belém, Pará, Brazil; ‡ Biological Engineering Laboratory, Guamá Science and Technology Park (PCT Guamá), Federal University of Pará, Perimetral da Ciência Avenue, km 01, s/n, Espaço Inovação Building, Guamá, 66075-750 Belém, Pará, Brazil; § Laboratory of Computer Modeling of Molecular Biosystems (CompMBio), 37871 Federal University of Pará, Bettina Ferro de Souza Street, Universitário, 66075-110 Belém, Pará, Brazil; ∥ 89124 Evandro Chagas Institute, Environment Section, Toxicology Laboratory, BR-316 Highway, km 7, s/n, Ananindeua, 67030-000 Belém, Pará, Brazil

## Abstract

Conventional water treatment typically employs chemical coagulants and flocculants, such as aluminum sulfate and iron salts. However, the intensive use of these products can lead to negative environmental impacts and pose significant health risks. In this context, bioflocculants derived from renewable sources, such as tannins extracted from açaí seeds (*Euterpe oleracea*), represent a promising alternative for sustainable production. This study investigated the efficiency of a cationic bioflocculant (BioTAC), developed from açaí seed tannins and chemically modified via the Mannich reaction, for the removal of toxic metals from aqueous media. Structural characterization of BioTAC was performed using mid-infrared Fourier transform spectroscopy (MIR-FTIR), and metal removal efficiency was assessed by inductively coupled plasma mass spectrometry (ICP-MS) at various bioflocculant concentrations (52–87 parts per billion, ppb) and pH values (4–10). The FTIR spectra revealed characteristic absorption bands in the ∼3300 cm^–1^ region, corresponding to O–H stretching, as well as peaks at ∼2930, ∼2880 cm^–1^, ∼1750 cm^–1^, and ∼1610 cm^–1^, and bands between 950 and 1150 cm^–1^. The high removal rates (91–100%) for aluminum (Al), titanium (Ti), iron (Fe), copper (Cu), zinc (Zn), and uranium (U) were achieved with concentrations between 69 and 87 ppb in an alkaline medium. Alkalinization was found to be crucial, as it promoted the deprotonation of the phenolic hydroxyl groups, thereby enhancing the chelation capacity for metal ions. Consequently, the application of BioTAC not only mitigates agro-industrial waste but also reduces dependence on chemical agents for treating water contaminated with toxic metals.

## Graphical abstract

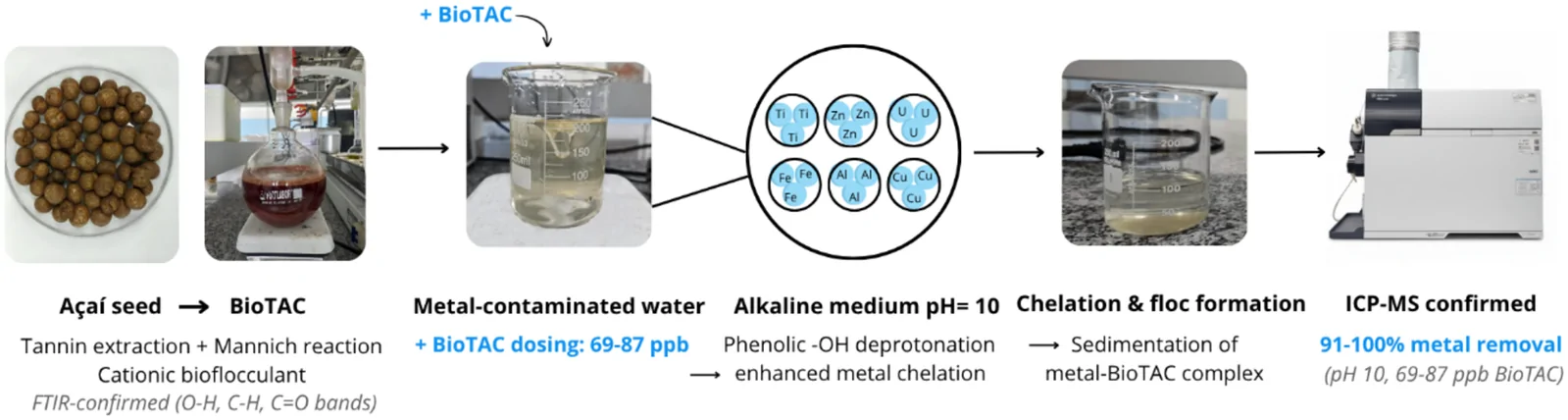

## Introduction

Water is one of the most abundant natural resources on Earth and is essential for human survival. In Brazil, approximately 4.8 million people still lack access to potable water.^1^ The country’s water and wastewater treatment is commonly performed using technologies based on chemical coagulants and flocculants. The most widely used of these include aluminum sulfate, iron salts, and synthetic polymers. Among these compounds, aluminum sulfate is widely used as a coagulant in drinking water and wastewater treatment due to its effectiveness, availability, and relatively low operational cost.^2,3^

However, the extensive use of chemical coagulants and flocculants can result in environmental impacts and potential risks to human health. The large-scale application of these compounds may contribute to the accumulation of residual metals and other chemical contaminants in treated water. Exposure to these substances has been associated with adverse health outcomes, including cancer associated with arsenic contamination and neurodegenerative diseases, such as Alzheimer’s disease and Parkinson’s disease, which have been linked to chronic exposure to aluminum and other neurotoxic metals.^3–7^

In this context, bioflocculants emerge as a promising and sustainable solution, exhibiting coagulant and flocculant properties similar to conventional agents but with a lower environmental impact. Tannins extracted from the seeds of açaí (*Euterpe oleracea*), an abundant agro-industrial byproduct in the Amazon region, demonstrate significant potential for water treatment applications.^8–12^ In recent years, tannin-based matrices have been extensively investigated for the remediation of highly toxic heavy metals. Among these studies, modified biopolymers derived from natural tannins have been successfully applied to the rapid removal of trace elements such as copper (Cu), chromium (Cr), and mercury (Hg), outperforming conventional inorganic coagulants under various pH conditions.^13,14^ Furthermore, tannin matrices have demonstrated remarkable chelating affinity for the selective recovery of critical metals from industrial effluents, driven by the strong complexation capacity of their abundant phenolic hydroxyl groups.^15^

Despite its promising potential, gaps remain in scientific knowledge regarding the production and application of a cationic bioflocculant derived from açaí seeds (BioTAC) in the removal of trace elements. Therefore, this study aims to address these gaps by evaluating the flocculation performance of the synthesized cationic bioflocculant (BioTAC) in the simultaneous removal of 20 trace elements from a multimetallic aqueous solution under different pH conditions.

## Materials and Methods

### Materials and Reagents

Açaí seeds were supplied by Guamá Polpas (Belém, PA, Brazil). A certified reference material (NIST, 1640a)^16^ was used to prepare simulated contaminated water containing 10 ppb of various metal ions, including Aluminum (Al), antimony (Sb), arsenic (As), barium (Ba), cadmium (Cd), chromium (Cr), copper (Cu), cobalt (Co), titanium (Ti), iron (Fe), lead (Pb), manganese (Mn), mercury (Hg), molybdenum (Mo), nickel (Ni), selenium (Se), uranium (U), vanadium­(V), rubidium (Rb), and zinc (Zn). All other reagents, such as formaldehyde, ammonium chloride, and hydrochloric acid, were of analytical grade (P.A.).

## Optimization of Tannin Extraction

### Raw Material Preparation

The seeds were initially washed and then dried in a forced-air oven at 65 °C for 24 h. The dried material was ground in a knife mill (Willey, Star FT 50/1) using a 10 mm sieve.

## Experimental Design

The extraction optimization was performed by using a 2^2^ full factorial design to evaluate the influence of temperature (*X*_1_) and time (*X*_2_) on the tannin yield. The operational parameters were kept constant in all analyses, including the use of distilled water as the solvent, a standardized particle size of 10 mm, and a solid-to-liquid ratio of 1:4 (w/v). The evaluated factors and their corresponding levels are presented in Table 1. The experimental matrix (Table 2) consisted of seven runs (four factorial points and three replicates at the center point) conducted in a random order.

**Table 1:** Factors and Levels Used in the Factorial Design

factors	code	–1	0	+1
temperature (°C)	*X*_1_	50	75	100
time (min)	*X*_2_	30	45	60

**Table 2:** Experimental Matrix of the Factorial Design

run	(*X*_1_)	(*X*_2_)	(*X*_1_ × X_2_)	temperature (*X*_1_)	time (*X*_2_)
1	–	–	+	50	30
2	+	–	–	100	30
3	–	+	–	50	60
4	+	+	+	100	60
5	0	0	0	75	45
6	0	0	0	75	45
7	0	0	0	75	45

Each extraction was conducted in sealed Erlenmeyer flasks by mixing 25 g of ground material with 100 mL of distilled water (1:4 w/v). The mixtures were kept in a water bath (Quimis Q215M1) under the conditions specified for each run (Table 2). At the end of each extraction, the extract was separated from the solid material via filtration. The resulting liquid extract was then analyzed for the quantification of condensed tannin content using the butanol-HCl method.^17^

### Production of BioTAC

The synthesis of the cationic bioflocculant based on açaí seed tannins (BioTAC) was performed via the Mannich reaction. The reaction parameters, stoichiometric ratios, temperatures, and reaction times were determined based on an adaptation of the method described by Velasco and Brasil, developed for the cationization of tannins from açaí waste. These specific conditions were selected to maximize the introduction of cationic amine groups into the polyphenolic matrix, while avoiding excessive crosslinking of the polymer.^18^ Initially, a cationizing iminium chloride solution was prepared by the sequential addition of 220 mL of formaldehyde, 58 g of ammonium chloride, and 28 mL of hydrochloric acid. The solution was heated to 80 °C with stirring (300 rpm) until the turning point was reached, at which point the temperature was reduced to 65 °C and maintained for 180 min. Subsequently, 298 mL of the tannin extract was added to 298 mL of the cationizing solution, and the resulting mixture was kept under agitation for 7 h to obtain BioTAC.

### Characterization of BioTAC by Mid-Infrared Fourier Transform Spectroscopy (MIR-FTIR)

The identification of the functional groups present in the bioflocculant was performed using mid-infrared spectroscopy (MIR) on an Agilent Cary 630 FTIR spectrometer (Santa Clara, CA, USA). The equipment operated with an attenuated total reflectance (ATR) module equipped with a zinc selenide (ZnSe) crystal and a deuterated triglycine sulfate (DTGS) detector.

Prior to analysis, BioTAC was subjected to a lyophilization process to ensure the complete removal of residual moisture while preserving the structural integrity of the polyphenols. The lyophilized material was deposited directly onto the crystal, and the spectra were obtained under the following conditions:

## Assessment of Metal Removal Efficiency

### Metal Removal Assays

The bioflocculant’s efficiency was assessed through flocculation assays using a 250 mL synthetic multimetallic solution. The solution was formulated with an individual concentration of 10 ppb for each of the following ions: aluminum (Al), antimony (Sb), arsenic (As), barium (Ba), cadmium (Cd), chromium (Cr), copper (Cu), cobalt (Co), titanium (Ti), iron (Fe), lead (Pb), manganese (Mn), mercury (Hg), molybdenum (Mo), nickel (Ni), selenium (Se), uranium (U), vanadium­(V), rubidium (Rb), and zinc (Zn).

A uniform concentration of 10 ppb for each metal was strategically selected to simulate a tertiary treatment stage. Removing contaminants at trace levels requires an active agent with exceptionally high chemical affinity, as conventional coagulants often exhibit reduced effectiveness at such low concentrations. Furthermore, while 10 ppb may comply with regulatory limits for some elements, this concentration exceeds the stringent maximum contaminant levels established by international drinking water guidelines for highly toxic elements like Hg, Cd, and Pb.^19^ The use of this concentration also aligns with the profile of the Certified Reference Material (NIST 1640a-Trace Elements in Natural Water) used to add the standard to the matrix and ensure the analytical accuracy of the inductively coupled plasma mass spectrometry (ICP-MS) measurements.^20^

BioTAC was evaluated at different concentrations (52, 69, and 87 ppb) and pH conditions (4, 7, and 10); the pH adjustments were monitored using universal pH indicator strips, and the flocculation assays were conducted using a digital benchtop stirrer (Bel Engineering AM-5) to ensure control of both rapid and slow mixing speeds (rpm). The experimental procedure, based on Lugo,^13^ involved three stages: a rapid mixing step (120 rpm for 2 min) to disperse the bioflocculant; a slow mixing step (50 rpm for 15 min) to promote floc agglomeration; and a 30 min settling period for floc sedimentation.

Three distinct groups were evaluated in triplicate. The first group served as a control, consisting of pure distilled water for the instrumental calibration. The second group was the tannin extract, which was analyzed to verify the presence of any constituent heavy metals, following the methodology of Da Costa.^21^ Finally, the third group comprised the treated samples, which involved adding BioTAC at different concentrations to the simulated multimetallic water to assess its efficacy in removing metal ions from the solution.

## Chemical and Statistical Analyses

### Quantification of Metals by ICP-MS

The samples were analyzed in triplicate by inductively coupled plasma mass spectrometry (ICP-MS, Agilent Technologies 7900). The analyses were performed on the basis of a calibration curve ranging from 1 to 160 ppb. The curve was prepared in a multimetallic mix matrix, and each point was analyzed in triplicate to ensure data reproducibility. Recovery tests were also conducted by adding the 1640a certified standard^16^ to contaminated samples to evaluate the analytical accuracy. The obtained recoveries were within the limits recommended by Standard Methods 3000/3125,^20^ thus validating the reliability of the results. The analyzed metals were the same as those previously described in the synthetic multimetallic solution. The limit of detection (LOD) and limit of quantification (LOQ) values are presented in Supporting Table S1.

## Statistical Analysis of the Optimization

Data from the Factorial Design were evaluated by analysis of variance (ANOVA) and Tukey’s test at a 95% significance level (*p* < 0.05), using the software. The results were fitted to a first-order polynomial model with interaction (eq 1)­

1 $$Y=\beta_{0}+\beta_{1}X_{1}+\beta_{2}X_{2}+\beta_{12}X_{1}X_{2}$$

where *Y* represents the condensed tannin yield (mg/mL), *X*_1_ and *X*_2_ are the coded values for temperature and time, respectively, and β are the regression coefficients.

## Results

### Optimization of Condensed Tannin Extraction

The optimization of tannin extraction from *E. oleracea* was performed by using a 2^2^ factorial design. Analysis of variance (ANOVA) indicated that the interaction between the factors temperature (*X*_1_) and time (*X*_2_) was statistically significant (*p* < 0.01). The condensed tannin yields obtained in each of the seven experimental runs are presented in Table 3.

**Table 3:** Condensed Tannin Yield (mg/mL) from Açaí Seeds According to the 2^2^ Factorial Design with a Central Point

	experimental conditions		
run	temperature (°C) (*X*_1_)	time (min) (*X*_2_)	condensed tannins (mg/mL)
1	50 (−1)	30 (−1)	33.58 ± 2.18
2	100 (1)	30 (−1)	31.87 ± 2.22
3	50 (−1)	60 (1)	31.67 ± 2.60
4	100 (1)	60 (1)	25.56 ± 1.45
5	75 (0)	45 (0)	33.04 ± 0.80
6	75 (0)	45 (0)	30.59 ± 0.99
7	75 (0)	45 (0)	33.30 ± 2.15

Statistical analysis using Tukey’s test (*p* < 0.05) and Figure 1 showed that the experimental condition of 50 °C for 30 min (run 1) resulted in the highest condensed tannin yield (33.58 ± 2.18 mg/mL). This value was statistically higher than the lowest yield, which was 25.56 ± 1.45 mg/mL, obtained under the 100 °C for 60 min condition. Based on these results, the 50 °C for 30 min condition was selected for the subsequent synthesis of BioTAC.

**Figure 1:**
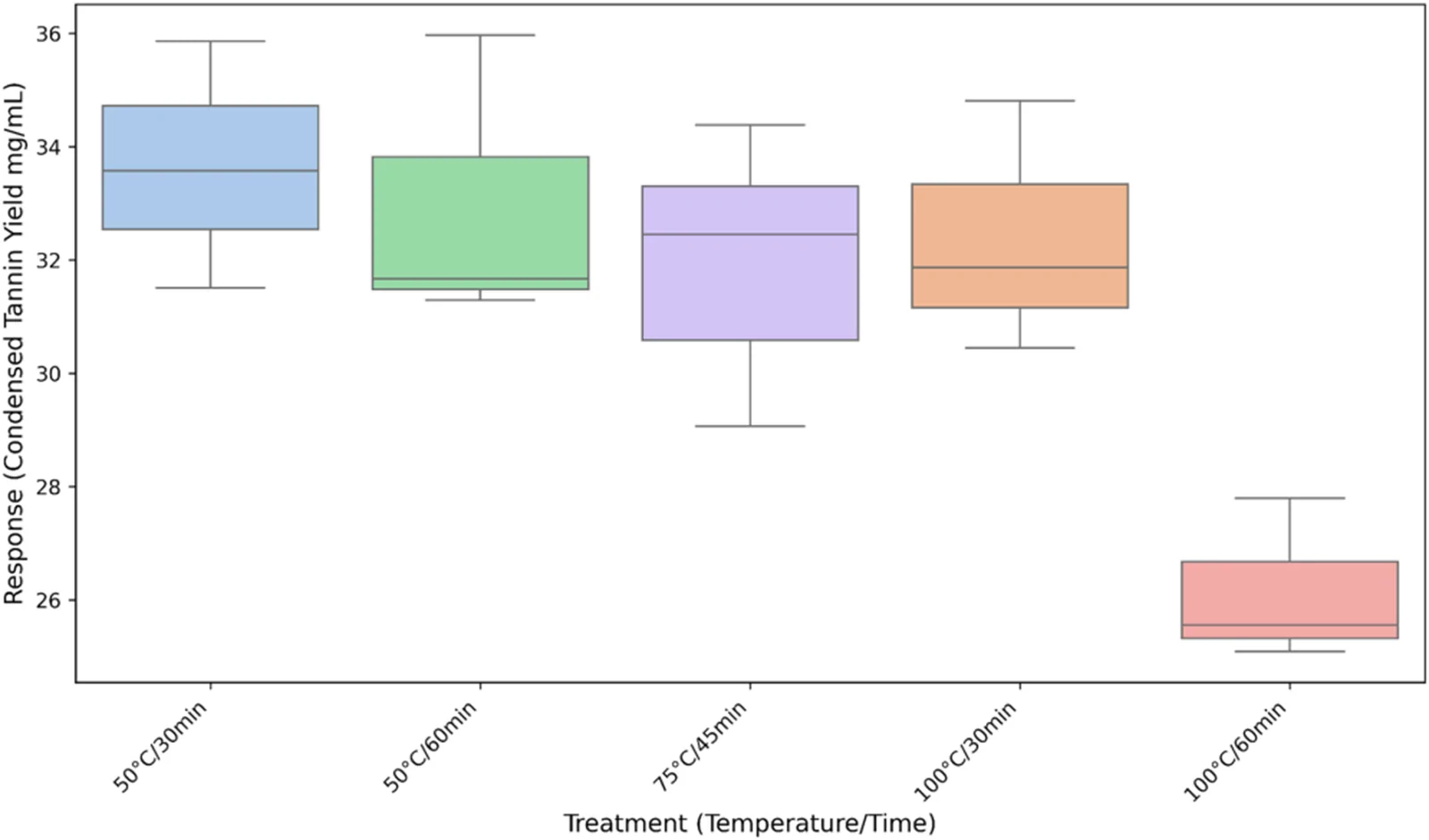
Box plot of the responses for the combinations of factors *X*_1_ and *X*_2_.

## Chemical Characterization by MIR-FTIR

Structural characterization of BioTAC by mid-infrared Fourier transform spectroscopy (MIR-FTIR) revealed the presence of functional groups that are determinant for its activity (Figure 2). The broad band centered at approximately 3400 and 3300 cm^–1^ reflects the axial stretching vibrations of hydroxyl (O–H) groups from alcohol structures intensely associated via intermolecular hydrogen bonding. In the 3100–2760 cm^–1^ range, a discrete peak with lower amplitude was identified between ∼2930 and ∼2880 cm^–1^, corresponding to the stretching of aliphatic C–H bonds.^22–25^

**Figure 2:**
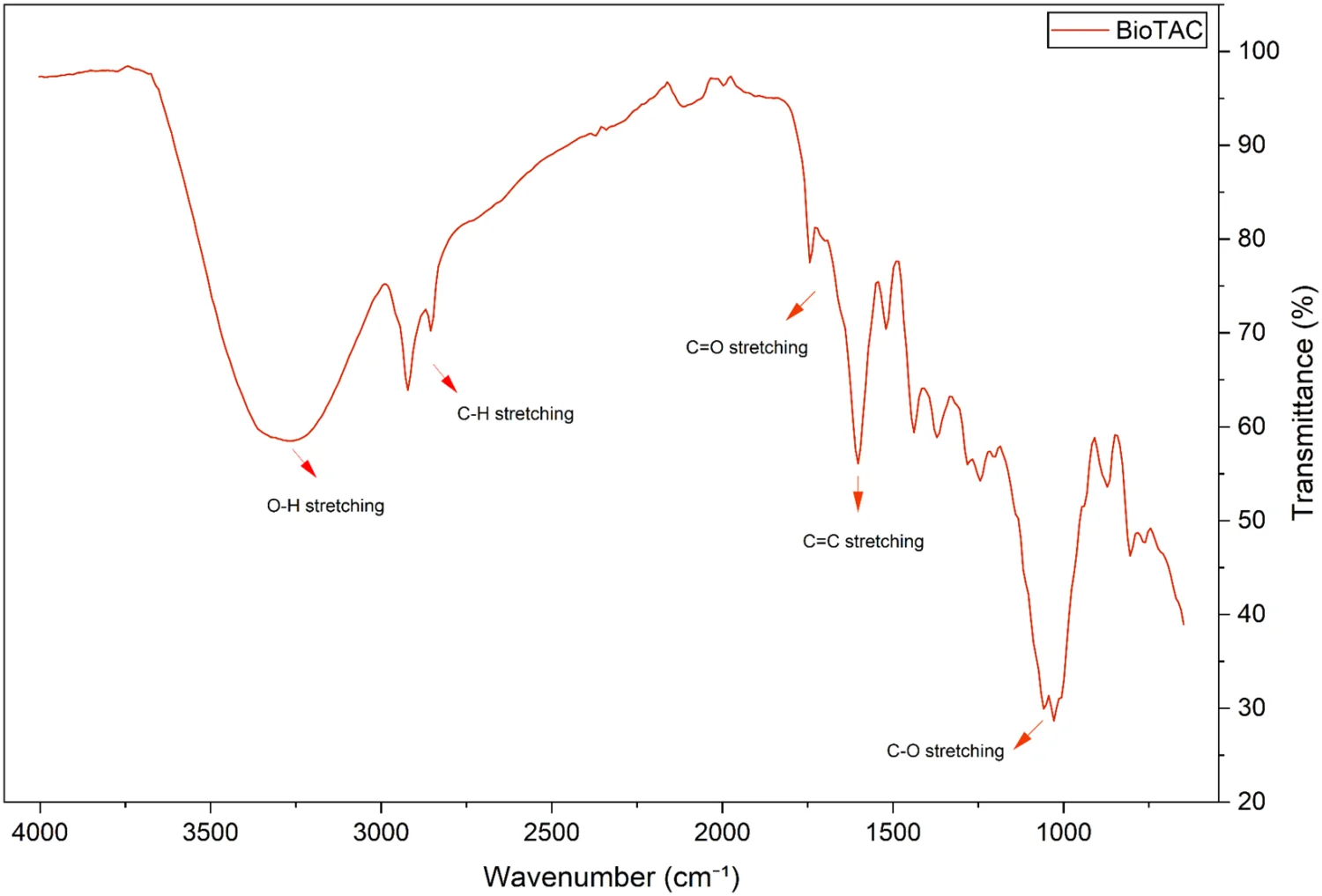
Mid-infrared spectroscopy of BioTAC.

In the region of oxygenated groups and carbon skeleton (1800–1000 cm^–1^), a characteristic signal was recorded at 1750 cm^–1^, attributed to carbonyl (C=O) stretching.^23–25^ Additionally, the absorption profile around 1610 cm^–1^ evidenced axial stretching vibrations of the C=C skeleton, confirming the presence of aromatic structures in the sample.^22,23,25^ Finally, the strong absorption zone between 1200 and 900 cm^–1^ indicated axial stretching profiles of phenolic C–O bonds at ∼1130 cm^–1^ and alcohol-associated C–O linkages at ∼911 cm^–1^,^22,23,27^ consolidating the macromolecular and polyfunctional nature of the material.

## Metal Removal Efficiency

### Control Assays

The inductively coupled plasma mass spectrometry (ICP-MS) analyses confirmed that the ultrapure water used in the experiments detected metal concentrations below the limits of detection (LOD). Meanwhile, the tannin extract revealed trace concentrations of V (0.0134 ppb), Mn (0.178 ppb), Zn (5.57 ppb), Rb (0.078 ppb), Mo (0.040 ppb), and Ba (0.031 ppb).

## Removal by Metal Group

At bioflocculant dosages of 52, 69, and 87 ppb across pH levels of 4, 7, and 10, no statistically significant reductions were observed for the elements As, Rb, and Sb. For these three metals, under specific conditions (notably pH 10 and pH 4), the final concentrations remained near or even exceeded initial levels.

The performance analysis of BioTAC for V, Mo, and Se indicated that the highest removal efficiencies were achieved at pH 4 (Figure 3). V and Mo showed average reductions of approximately 33 and 40%, respectively, at the Bio II (69 ppb) and Bio III (87 ppb) concentrations, while Se removal was more significant, reaching approximately 52% at pH 4. Conversely, an increase in concentrations was observed for these three metals when treated at pH values of 7 and 10, indicating the bioflocculant ineffectiveness in neutral and alkaline media.

**Figure 3:**
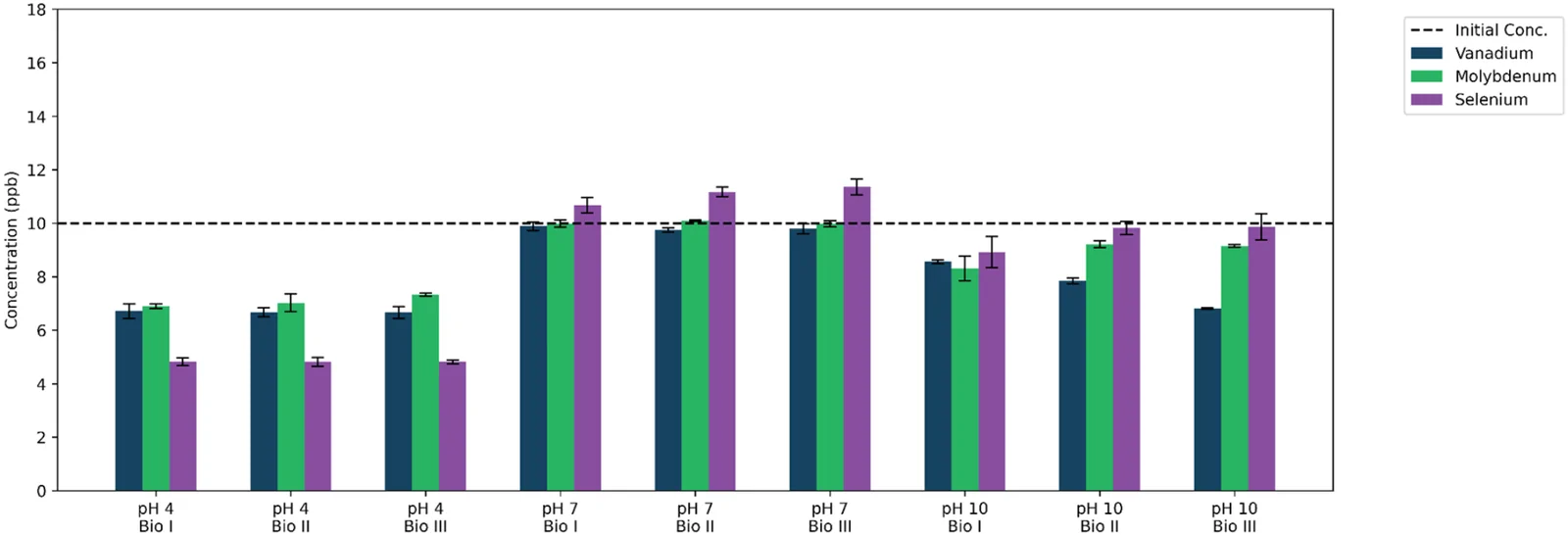
Reduction in the concentrations of vanadium, molybdenum, and selenium after treatment with BioTAC under different pH and concentration conditions.

Legend: Residual concentrations of vanadium, molybdenum, and selenium after the treatment of contaminated water with different bioflocculant dosages (Bio I—52 ppb, Bio II—69 ppb, Bio III—87 ppb) under acidic (pH 4), neutral (pH 7), and alkaline (pH 10) conditions.

The analysis of Mn removal demonstrated higher BioTAC efficiency at higher pH values (Figure 4). The maximum removal rate was observed at pH 10, with an efficiency of 80.8%, followed by a 50.6% reduction at pH 7. It is noteworthy that this efficacy was achieved with the BioTAC concentration corresponding to Bio II (69 ppb), indicating that the flocculation process is dependent on both pH and the agent’s dosage.

**Figure 4:**
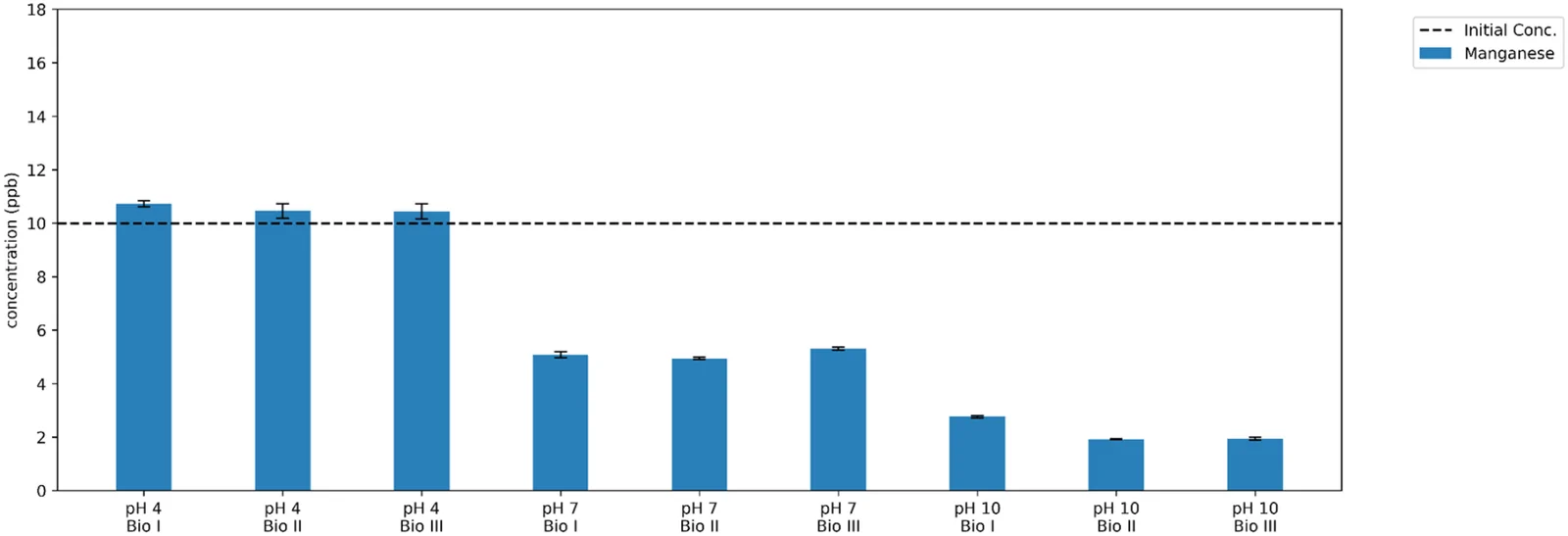
Effect of pH and BioTAC concentration on the reduction of manganese in an aqueous medium.

Legend: Residual manganese (Mn) concentration in contaminated water following treatment with three bioflocculant concentrations (Bio I—52 ppb, Bio II—69 ppb, and Bio III—87 ppb) under acidic (pH 4), neutral (pH 7), and alkaline (pH 10) conditions.

The metals Cr, Co, Ni, Cd, and Pb showed removal efficiencies between 85 and 89% at pH 10 with the highest dosage of Bio III (87 ppb) (Figure 5). Specifically, the reduction was 88.6% for Cr, 87.9% for Co, 89.6% for Ni, 87.1% for Cd, and 86.5% for Pb. Ba exhibited 87.8% removal with the lowest concentration (Bio I—52 ppb) at pH 10, and 85% removal at pH 7 with the use of Bio III (87 ppb).

**Figure 5:**
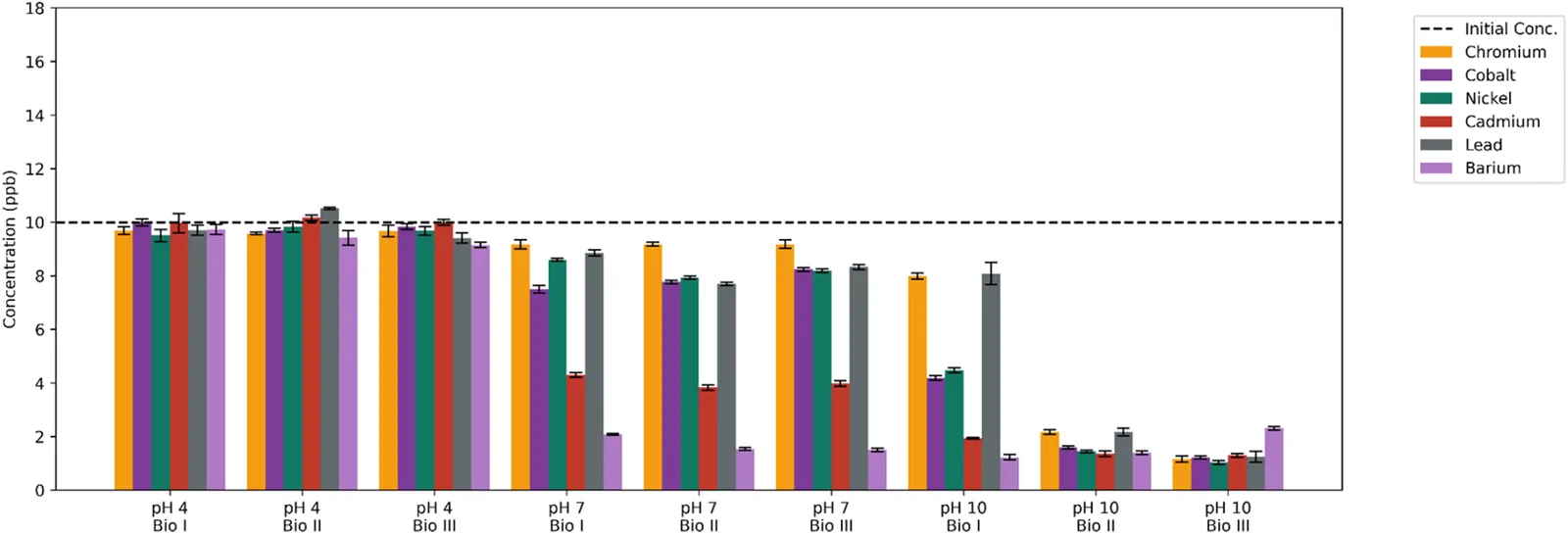
Removal of Cr, Co, Ni, Cd, Pb, and Ba by treatment with BioTAC.

Legend: Residual concentrations of chromium (Cr), cobalt (Co), nickel (Ni), cadmium (Cd), lead (Pb), and barium (Ba) after treatment of the contaminated water with the BioTAC bioflocculant, under different pH conditions (acidic—4, neutral—7, and alkaline—10) and concentrations (Bio I—52 ppb, Bio II—69 ppb, and Bio III—87 ppb).

The chemical elements that showed reductions between 91 and 100% were Al, Ti, Fe, Cu, Zn, and U, especially at pH 10 (Figure 6). Al, Fe, and Zn reached 100% removal with the Bio II and Bio III concentrations. The elements Ti, Cu, and U achieved high removal efficiencies with the maximum concentration (Bio III—87 ppb), with removals of 93.5% for Ti and 91.6% for U.

**Figure 6:**
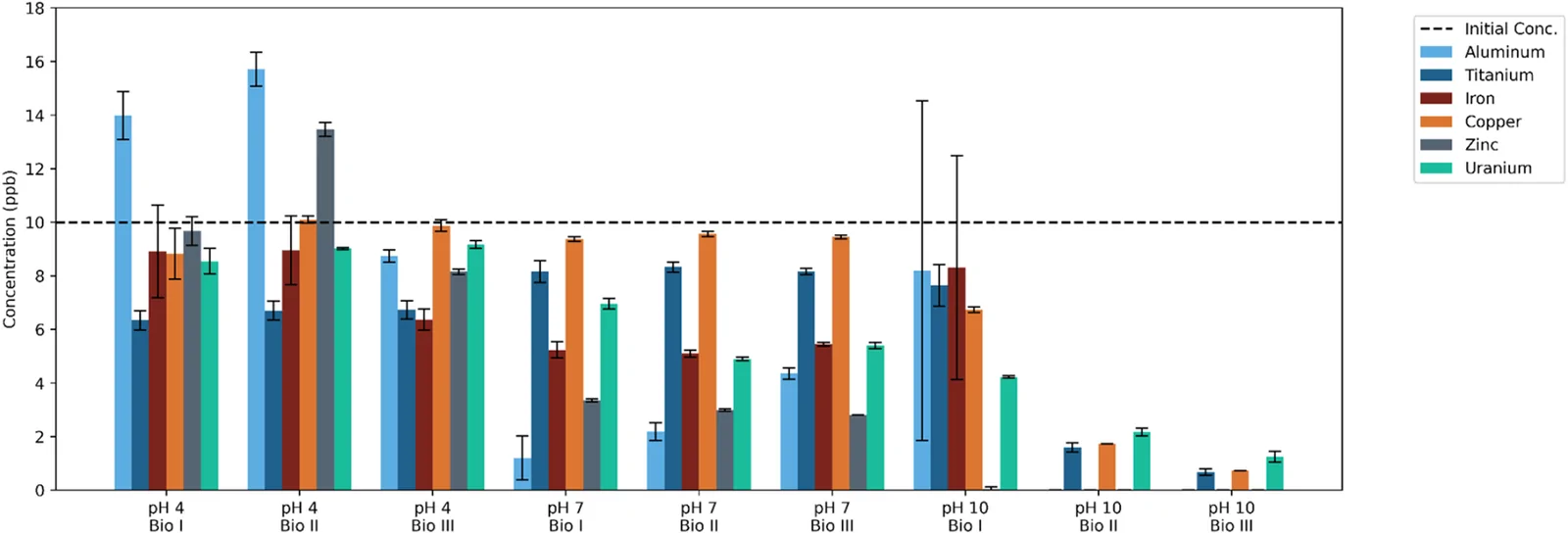
Removal of Al, Ti, Fe, Cu, Zn, and U after treatment with BioTAC.

Legend: Residual concentrations of aluminum (Al), titanium (Ti), iron (Fe), copper (Cu), zinc (Zn), and uranium (U) in contaminated water, after treatment with the BioTAC bioflocculant at three dosages (Bio I—52 ppb, Bio II—69 ppb, and Bio III—87 ppb) under different pH conditions (4, 7, and 10).

Acidic and neutral pH conditions yielded higher efficiencies for Hg removal, with reductions ranging from 41 to 53.4% when using the lowest dosage (Bio I—52 ppb) (Figure 7). An increase in the bioflocculant concentration led to reduced efficiency. At alkaline pH, mercury showed a significant increase in concentration.

**Figure 7:**
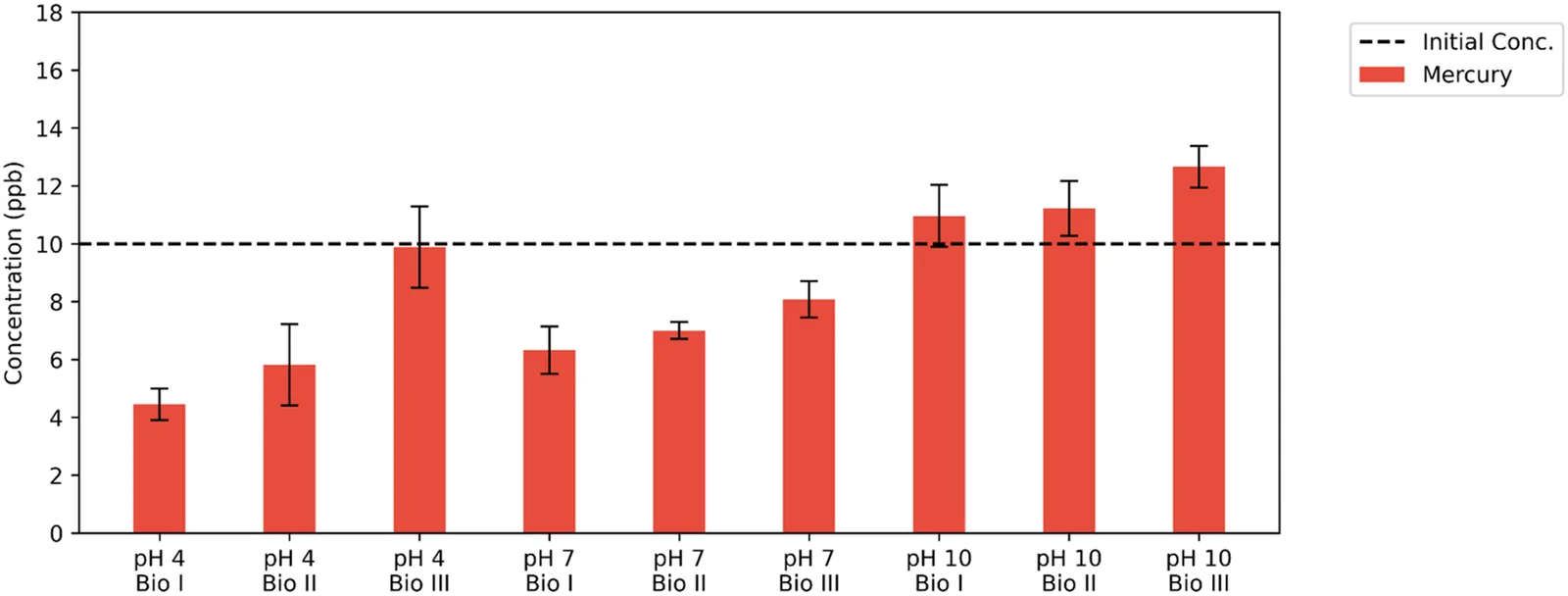
Removal of mercury (Hg) by treatment with BioTAC.

Legend: Residual concentration of mercury (Hg) in contaminated water after treatment with the BioTAC bioflocculant, applied at three concentrations (Bio I—52 ppb, Bio II—69 ppb, and Bio III—87 ppb) under different pH conditions (acidic—4, neutral—7, and alkaline—10).

## Discussion

In the ∼3400 and ∼3300 cm^–1^ region, the axial stretching vibrations correspond to the hydroxyl (O–H) groups of condensed polyphenols associated via intermolecular hydrogen bonding. This profile indicates a high degree of polymerization of the tannins, characterized by the convergence of hydroxyl groups at different molecular positions. The prevalence of these phenolic −OH groups, widely known for their strong reducing capacity, is in close consonance with the high content of condensed tannins detected in the extract used for the bioflocculant synthesis.^22–24^ Narrow peaks at ∼2930 and ∼2880 cm^–1^, corresponding to the stretching of aliphatic C–H bonds.^22,23^ This characteristic, observed in the present study, may suggest methylation in the chemical structure of the compounds.^24,25^ The band at ∼1750 cm^–1^ is characteristic of the carbonyl group (C=O), indicating C=O stretching vibrations of free gallic acid in hydrolyzable tannins.^23,25^ This serves as a specific marker for gallotannins when accompanied by bands at ∼870 cm^–1^.^26^ The region around ∼1610 cm^–1^ indicates C=C stretching vibrations of aromatic rings, which are abundant in tannins composed essentially of aromatic compounds.^22,23,25^ Finally, bands between 950 and 1150 cm^–1^ indicate phenolic C–O and heterocyclic C–O–C stretching.^22,23^

Research by Ricci et al.^27^ identified peaks between 1290 and 1150 cm^–1^ as characteristic of hydrolyzable tannins. According to the literature, this vibrational splitting arises from the combination of C–O stretching and hydroxyl (OH) deformation modes associated with the carboxylic acid (COOH) functional group. Thus, this spectral range acts as a diagnostic molecular fingerprint for hydrolyzable tannins, such as gallic acid and tannic acid (gallotannin).

The tannin extraction yield was significantly influenced by the interaction between temperature and time, indicating that these factors do not operate independently. The greater extraction efficiency observed at moderate temperatures and shorter contact times is likely due to the thermal degradation of polyphenolic compounds under more severe conditions.^28^ Previous studies corroborate these findings. Naima^29^ and Bianchi^30^ reported similar trends, observing a reduction in extraction yield when the temperature exceeded approximately 60 °C, a threshold consistent with recent optimization studies.^28^ This behavior has also been reported specifically for tannins extracted from açaí seeds. Furthermore, the hot water extraction methodology reported by Bianchi^30^ identified such extracts as promising precursors for resin formulation and heavy metal remediation agents, owing to the high chemical reactivity of extracted condensed tannins.^31^

The detection of metals in the raw tannin extract can be attributed to the natural presence of minerals in the soil of the açaí palm (*E. oleracea*) cultivated areas, classified as essential macronutrients and micronutrients. The presence of elements such Ba and Rb, classified as toxic trace elements, may be related to the geochemical characteristics of soils near mining regions.^32–34^ While Ba generally exhibits low solubility, it can become an environmental contaminant at high saturation levels.^32^ Rb, acting as an analog of potassium, can be competitively absorbed by the plant, potentially compromising growth.^35^ Despite their detection, the levels found in the extract were low and do not pose a toxicity risk for its intended application as a bioflocculant.

The flocculation mechanism of BioTAC involves a synergistic combination of charge neutralization, polymer bridges, and chemical chelation. The cationic reaction introduces amine groups into the polymer structure, facilitating the destabilization of suspended particles through charge neutralization and the formation of intermolecular bridges.^13,18^ Furthermore, the high efficiency in removing heavy metals, especially under alkaline pH conditions, is driven by the chemical structure of the condensed tannins.^11,14^ The abundant phenolic hydroxyl groups present in the tannin matrix undergo deprotonation at higher pH levels. This process increases the local electron density, thereby enhancing the chelation of multivalent metal ions and promoting the formation of insoluble metal–tannin complexes that readily precipitate.^18,36^

In the remediation assays, the low initial efficiency indicated that the BioTAC mechanism was not sufficiently effective for these elements at tested dosages. The lack of removal for Sb and As, even at pH levels considered ideal in the literature for ferric coagulants,^37–40^ highlights a different mechanism for this bioflocculant. The same occurred with Rb, for which a neutral pH^41^ was insufficient. The increase in the final concentrations of these three elements suggests the possible release of metallic compounds previously associated with the bioflocculant matrix, which may have contributed to their increased levels.

The higher removal efficiency for V, Mo, and Se at pH 4 indicates that using BioTAC in an acidic medium favors interaction with these metals, as under acidic conditions they exhibit greater ionic solubility and reactivity with the tannin’s phenolic groups. The increased concentration at higher pHs may indicate adsorption instability or compound release. For V, the result corroborates Bello,^42^ who also observed a drop in efficiency at pH 10. For Mo, removal at an acidic pH is consistent with its physicochemical behavior, as at higher pHs it forms highly soluble molybdate anions (MoO_4_^2–^) with low affinity for tannins. Industrial literature points to alternative techniques, such as flotation, for effective Mo remediation.^43,44^ For Se, the ideal pH range between 4 and 6 reported by Hansen^45^ and Inam^46^ is reinforced by this study, and the instability at higher pHs may be related to the different ionic forms of selenium in solution.

The efficiency for Mn at pH 10 and 7 indicates an optimal range, as Mn tends to form more soluble, easily removable species, such as Mn­(OH)_2_ or MnO_2_, at alkaline pH. The maximum efficiency was observed with intermediate dosages (Bio II). The significant decrease in removal efficiency at higher bioflocculant dosages (Bio III) can be attributed to the phenomena of colloidal restabilization and steric hindrance. As a cationic polymer, an excessive dosage of BioTAC saturates the available binding sites on the target particles, causing a charge reversal from neutral or negative to highly positive. This charge reversal induces electrostatic repulsion of the complexes.^47,48^ Furthermore, an excess of polymer chains physically blocks the active sites, causing steric hindrance that prevents the formation of polymer bridges between distinct particles, hindering sedimentation as they are dispersed in the aqueous medium.^18,49^ The data reinforce the idea that each metal requires specific parameters for efficient removal.

The moderate efficiency for Cr, Co, Ni, Cd, and Pb at pH 10 indicates a strong affinity of these metals for tannins in an alkaline medium, where there is less competition with H^+^ ions and a greater tendency to form complexes or hydroxides. The results for Cr (88.6%), Co (87.9%), and Cd (87.1%) corroborate literature data that reported reductions with tannin-based flocculants of 87, 84.19, and 87%, respectively.^18,49,50^ The removal of Ni (89.6%) was superior to the 75% reported by Beltrán-Heredia,^51^ and the removal of Pb (86.5%) can be explained by the coordination between the metal and hydroxyl groups.^52^ The complex behavior of Ba, sensitive to both dosage and pH, suggests a more intricate interaction mechanism.

The high efficiency for Al, Ti, Fe, Cu, Zn, and U at alkaline pH demonstrates the bioflocculant’s high performance. The results for Al and Fe corroborate de Oliveira,^53^ who reported removals of 93.76 and 97.37% with a modified tannin, whereas BioTAC showed superior efficiency with 100% reduction. The performance for Zn was superior to that of Beltrán-Heredia,^51^ who obtained 70–75% reduction with modified tannins. For Ti, the data are similar to Righetto,^54^ who reported reductions close to 90%. The high removal of Cu at pH 10 occurs because the phenolic groups do not compete with active chelating groups, unlike at acidic pH.^55^ The U removal efficiency achieved by BioTAC was comparable to that reported by Sharma^56^ for a tannic-acid-modified nanomagnetite adsorbent, highlighting its potential as an effective material for radionuclide removal from aqueous solutions.

The behavior of Hg, with better removal at acidic/neutral pH and low dosages, and redissolution at alkaline pH, can be explained by Pearson’s hard and soft acids and bases (HSAB) theory.^57^ The lower efficiency for Hg (a soft acid) in alkaline media suggests a preference for ligands with lower electronegativity, which are less available when hydroxyl ions (hard bases) dominate the medium. This finding corroborates the study by Lugo,^13^ which also highlighted better efficiency at neutral pH and the importance of reduced doses for Hg removal.

BioTAC exhibits significant variation in optimal pH conditions for the removal of different metal groups; uniform optimization in real-world mixed-pollution systems presents a practical challenge. To address this issue, future studies aimed at industrial applications should consider a collaborative multiparametric optimization. This sequential strategy may maximize the overall broad-spectrum efficiency of BioTACs in the treatment of complex effluents.

## Conclusion

BioTAC demonstrated high efficacy in the selective removal of various heavy metals, through a mechanism combining charge neutralization and optimized metal chelation by ionized phenolic hydroxyl groups. This environmentally friendly and low-cost alternative is a promising solution for treating contaminated water, with the potential to benefit vulnerable communities. However, it is essential to consider the bioflocculant’s selectivity towards different metals and pH ranges. Future research should focus on further characterizing the post-flocculation solids, evaluating its application to real industrial wastewater, investigating its integration with other treatment technologies, and conducting toxicological assessments to ensure the efficacy and safety of the process under practical operating conditions.

## Supplementary Material



## Notes

Limits of detection (LOD) and quantification (LOQ), expressed in parts per billion (ppb), for the analysis of the investigated metallic elements; LOD range: 0.09 ppb (cadmium) to 3.03 ppb (aluminum), LOQ range: 0.3–10 ppb, establishing the sensitivity parameters necessary for validating the instrumental method used in the study’s quantification of contaminants and micronutrients (PDF)
