# Supplementary material for: Optimizing the Production of a Condensed Tannin-Based Bioflocculant for Toxic Metal Remediation
Source: ACS Omega. 2026 Sep 13;11(38):57016–25. doi: 10.1021/acsomega.6c04045 (PMC13629034; doi:10.1021/acsomega.6c04045)
Supplement: Supplementary file 1 [file ao6c04045_si_001.pdf]

OPTIMIZING THE PRODUCTION OF A CONDENSED TANNIN-BASED BIOFLOCCULANT FOR TOXIC METAL REMEDIATION

Sara Raissa Lobato Ferreira[a]*, Jéssica Lyssa Araújo[a], Camilla Mariane Moura de Souza[b], Carlos Gabriel da Silva de Souza[c], Kleber Raimundo Freitas Faial[d], Neirivaldo Cavalcante da Silva[a], Nilton Akio Muto[a].

[a] Center for Valorization of Bioactive Compounds of the Amazon (CVACBA), Guamá Science and Technology Park (PCT Guamá), Federal University of Pará, Perimetral da Ciência Avenue, s/n, Espaço Inovação Building, Guamá, 66075-750 Belém, Pará, Brazil

[b] Biological Engineering Laboratory, Guamá Science and Technology Park (PCT Guamá), Federal University of Pará, Perimetral da Ciência Avenue, km 01, s/n, Espaço Inovação Building, Guamá, 66075-750 Belém, Pará, Brazil

[c] Laboratory of Computer Modeling of Molecular Biosystems (CompMBio), Federal University of Pará, Bettina Ferro de Souza Street, Universitário, 66075-110 Belém, Pará, Brazil

[d] Evandro Chagas Institute, Environment Section, Toxicology Laboratory, BR-316 Highway, km 7, s/n, Ananindeua, 67030-000Belém, Pará, Brazil

* Email: sara.ferreira@icb.ufpa.br

Table S1 - Limits of Detection (LOD) and Limits of Quantification (LOQ) for the trace metals analyzed by ICP-MS.

Metal	Symbol	LOD (ppb)	LOQ (ppb)
Aluminum	Al	3.03	10
Antimony	Sb	0.303	1
Arsenic	As	0.303	1
Barium	Ba	1.515	5
Cadmium	Cd	0.091	0.3
Chromium	Cr	1.515	5
Cobalt	Co	1.515	5
Copper	Cu	0.303	1
Iron	Fe	1.515	5
Lead	Pb	0.303	1
Manganese	Mn	1.515	5
Mercury	Hg	0.01	0.05
Molybdenum	Mo	0.303	1
Nickel	Ni	1.515	5
Rubidium	Rb	1.515	5
Selenium	Se	0.303	1
Uranium	U	0.303	1
Vanadium	V	1.515	5
Zinc	Zn	1.515	5
